# Overlapping anti-NMDAR encephalitis and multiple sclerosis: A case report and literature review

**DOI:** 10.3389/fimmu.2023.1088801

**Published:** 2023-01-30

**Authors:** Pan Liu, Hui Yan, Haizhe Li, Chunhua Zhang, Yanfang Li

**Affiliations:** ^1^ Department of Neurology, The Central Hospital of Shaoyang, Shaoyang, Hunan, China; ^2^ Department of Clinical Medicine, Xiangnan University, Chenzhou, Hunan, China

**Keywords:** anti-NMDAR encephalitis, multiple sclerosis, demyelinating disease, overlap, mycophenolate mofetil

## Abstract

Anti-N-methyl-D-aspartate receptor (NMDAR) encephalitis is an autoimmune-mediated disease characterized by complicated neuropsychiatric symptoms and the detection of cerebrospinal fluid antibodies against the GluN1 subunit of the NMDAR. With the proposed clinical method, more anti-NMDAR encephalitis patients have been discovered since its first report. However, anti-NMDAR encephalitis overlapping with multiple sclerosis (MS) is rare. Herein we report a male patient with anti-NMDAR encephalitis who developed MS in mainland China. Furthermore, we summarized the characteristics of patients who were diagnosed with overlapping MS and anti-NMDAR encephalitis in previous studies. Additionally, we pioneered the use of mycophenolate mofetil in immunosuppressive therapy, providing a novel therapeutic alternative for overlapping anti-NMDAR encephalitis and MS.

## 1 Introduction

Anti-N-methyl-D-aspartate receptor (NMDAR) encephalitis is the most common autoimmune-mediated encephalitis characterized by the manifestation of abnormal behavior, psychosis, seizures, abnormal movement, insomnia, and irritability (1). A few studies reported anti-NMDAR encephalitis overlapping with neuromyelitis optica spectrum disorders, acute demyelinating encephalomyelitis, and myelin oligodendrocyte glycoprotein (MOG) inflammatory demyelinating disease (2, 3). Nevertheless, anti-NMDAR encephalitis associated with multiple sclerosis (MS) is rare. Therefore, in this study, we report a case of a male patient with the course from anti-NMDAR encephalitis to MS in mainland China. Moreover, we concluded the clinical features of the patients with overlapping MS and anti-NMDAR encephalitis. Furthermore, we pioneered the use of mycophenolate mofetil for immunosuppressive therapies, which provides a new treatment option for overlapping anti-NMDAR encephalitis and MS.

## 2 Case report

**Figure 1:**
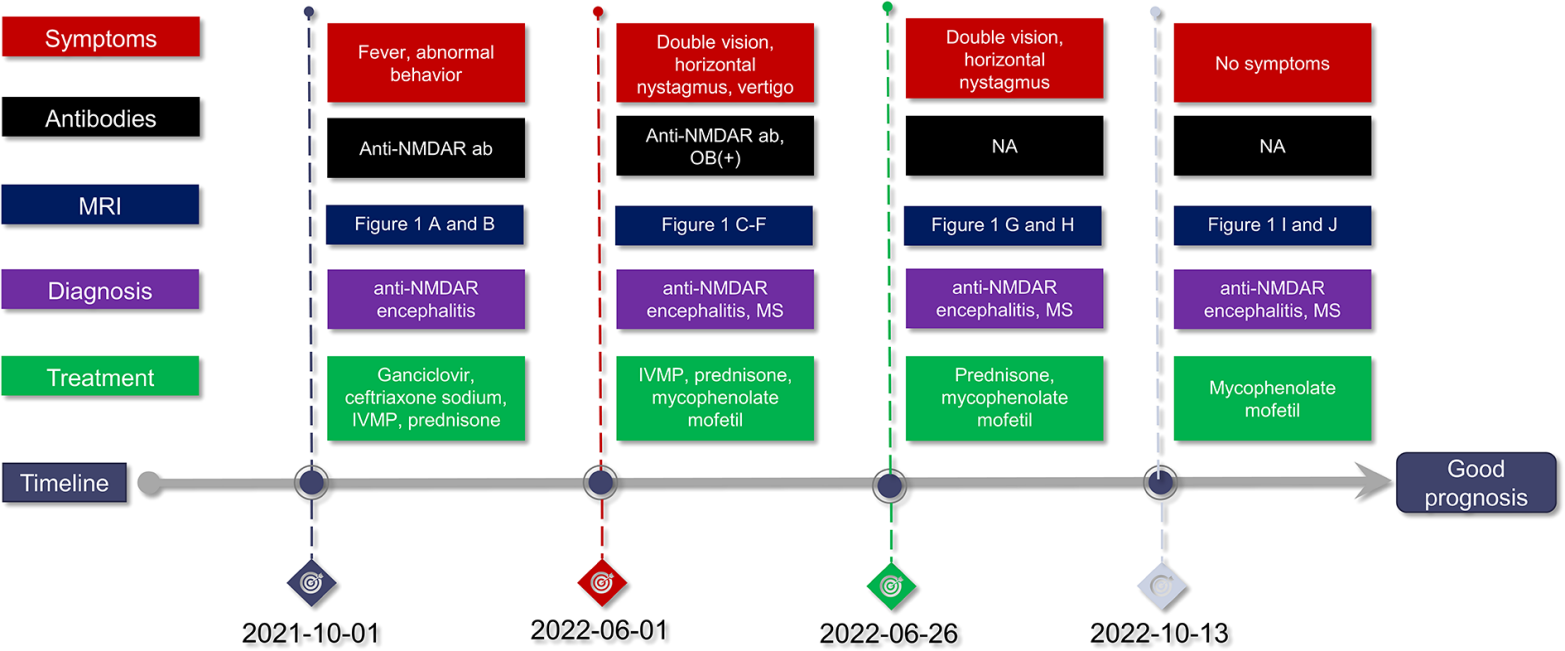
Disease duration in this case. Anti-NMDAR encephalitis, anti-N-methyl-D-aspartate receptor encephalitis; anti-NMDAR ab, anti-N-methyl-D-aspartate receptor antibody; MRI, magnetic resonance imaging; IVMP, intravenous methylprednisolone; MS, multiple sclerosis; NA, not available; OB, oligoclonal immunoglobulin bands.

**Figure 2:**
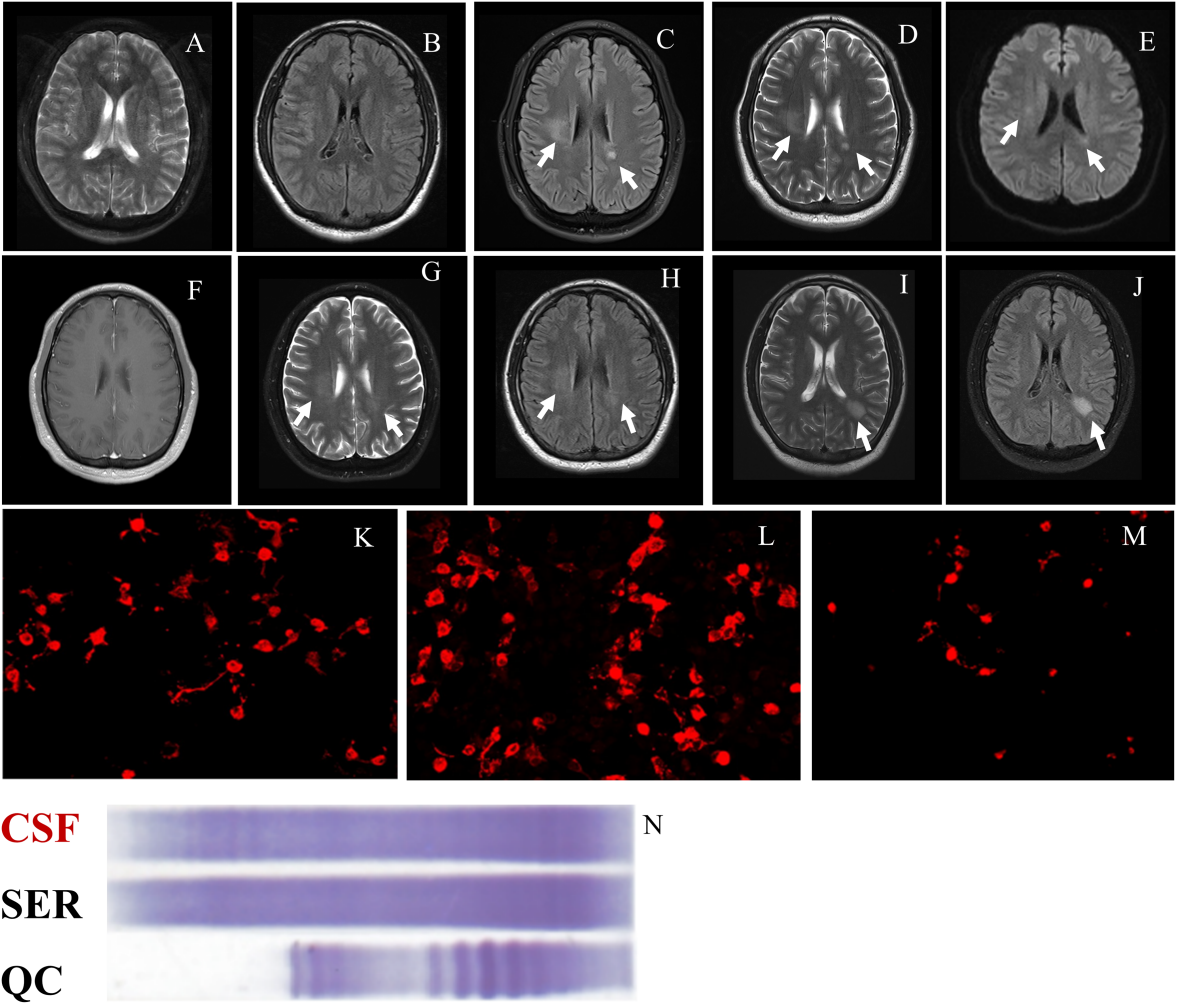
Brain MRI and antibodies in this case. **(A)** and **(B)** The MRI performed in October 2021 demonstrated normal results on T2 weight imaging and FLAIR imaging. **(C–E)** The MRI result showed significant hyperintensity in the right semioval center and the left posterior horn of the lateral ventricle on FLAIR imaging, T2 weight imaging, and DWI on June 1, 2022. **(F)** Enhanced magnetic resonance imaging performed on June 1, 2022, not showing a significant lesion. **(G)** and **(H)** The results of the MRI performed on June 1, 2022 demonstrated that the hyperintensity lesions were alleviated than before on T2 weight imaging and FLAIR imaging. **(I)** and **(J)** The results of the MRI performed on October 1, 2022 showed a new lesion with hyperintensity in the left posterior horn of the lateral ventricle on T2 weight imaging and FLAIR imaging. **(K, L)** Anti-NMDAR antibodies were found to be positive in serum (1:100) and in CSF (1:30) on June 1, 2022. **(M)** Anti-NMDAR antibodies were found to be positive in CSF (1:10). **(N)** OB was positive in CSF (counts, ≥2). CSF, cerebrospinal fluid; DWI, diffusion-weighted imaging; FLAIR, fluid-attenuated inversion recovery; MRI, magnetic resonance imaging; OB, oligoclonal immunoglobulin bands; anti-NMDAR, anti-N-methyl-D-aspartate receptor; QC, quality control; SER, serum.

**Figure 3:**
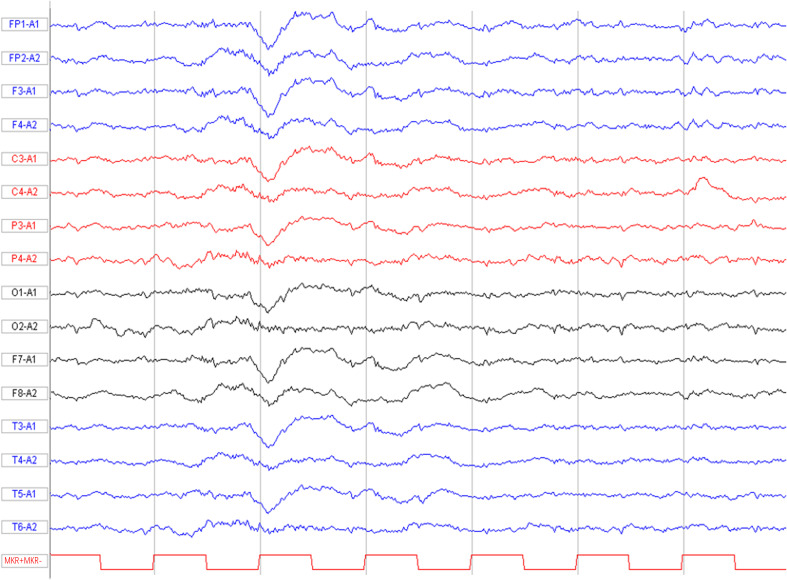
Basal EEG (recorded on day 7 from symptom onset). “Extreme delta brush” pattern with diffuse delta slowing and overriding high-frequency activity. EEG, electroencephalogram.

A graphical presentation of this patient is provided in **Figure 1**. At the age of 30 years old, a Chinese male patient was brought to the local hospital on October 1, 2021 because of abnormal behavior, which presented as obscene behavior towards women, aggressive behavior, and self-mutilation. In the past week, the patient suffered a fever with temperature of over 38°C after swimming. After taking ibuprofen, the temperature of the patient returned to normal. However, on September 28, 2021, the patient suddenly left their home and harassed young female individuals in public places. Then, the patient was taken by a policeman to a local psychiatric hospital due to his abnormal behavior. The various abnormal behaviors (including aggressive behavior and self-mutilation behavior) lasted for several minutes and were terminated before reaching the hospital. The computed tomography scans of the head and lungs were found to be normal. A blood routine examination revealed increased white blood cell counts (18.3 × 10^9^/L) and a high neutrophil percentage (87.4%). Moreover, a high C-reactive protein level (27.46 mg/L) was measured in the patient. Then, the patient was brought to the department of emergency in our hospital. A neurological examination revealed cervical rigidity. Muscle strength, muscle tension, tendon reflex, sensory nervous system, cerebellar functions, and Babinski signs were observed to be in the normal range. The magnetic resonance imaging (MRI) result of the brain turned out normal (**Figures 2A, B**). An electroencephalogram image of the patient showed an “extreme delta brush” pattern (**Figure 3**). A lumbar puncture was performed subsequently, by which slightly increased intracranial pressure (180 mmH_2_O) and high white cell counts (100 × 10^6^/L) were found, but the glucose and chloride levels of the cerebrospinal fluid (CSF) were normal. He was initially diagnosed with viral meningoencephalitis. Hence, he was administered with empirical intravenous anti-viral (ganciclovir, 500 mg/day, Q12h) and antibiotic (ceftriaxone sodium, 2,000 mg/day) therapy. After 1 day, the patient was transferred from the emergency department to the department of neurology. After the anti-viral and antibiotic treatment for 4 days, the indicators of infection in CSF decreased (white cell counts, 58 × 10^6^/L), but the above-mentioned symptoms were not alleviated. We suggested that the patient might have developed autoimmune encephalitis after the viral encephalitis due to trigger effects (4). Therefore, anti-neural antibodies in the blood and CSF were detected. Upon searching for antibodies (anti-NMDAR, anti-LGI1, anti-CASPR2, anti-AMPA1, anti-AMPA2, and anti-GABABR), the blood (1:100) and CSF (1:30) were found positive with anti-NMDAR antibodies by cell-based assay (CBA) (Kingmed Diagnostics Co., Ltd., which is the largest College of American Pathologists-certified laboratory in China) (PMID: 33106361) (**Figures 2K, L**). The patient was on intravenous methylprednisolone (IVMP) for 5 days (1,000 mg/day). Then, the glucocorticoid dose was gradually reduced to 60 mg/day and changed to oral prednisone maintenance. After the immunosuppressive treatment, the symptoms of behavioral abnormalities of the patient were gradually alleviated. On October 21, routine and biochemical tests of CSF were performed again, in which the white cell counts (30 × 10^6^/L) and intracranial pressure (100 mmH_2_O) were decreased. The patient was continued on the immunosuppressive treatment with prednisone (60 mg/day) after discharge (the dosage was reduced to 5 mg every 2 weeks, and the total immunosuppressive treatment time was approximately 6 months).

The patient was presented to our department on June 1, 2022 due to symptoms of diplopia and vertigo that lasted for 2 months. The symptoms of diplopia occurred when the patient looked far away and gazed to the left. The neurological examination discovered horizontal and vertical nystagmus on both eyes and a positive Romberg sign. The results of other neurological physical examinations were observed to be in the normal range. Electronystagmography was performed to examine the nystagmus in the patient. The results of electronystagmography showed spontaneous vertical nystagmus. Moreover, horizontal nystagmus was also observed using electronystagmography when both eyes gaze to the left or right side. The above-mentioned results suggested that the patient had internuclear ophthalmoplegia. Moreover, the patient stopped the immunosuppressive treatment since April 2022. Considering the previous history of the disease, we initially hypothesized that diplopia and nystagmus were the new symptoms of anti-NMDAR encephalitis. The CSF was positive (1:10) with anti-NMDAR antibodies (**Figure 2M**), but the antibody titer was lower than before. Moreover, the brain MRI result showed significant hyperintensity in the right semioval center and the left posterior horn of lateral ventricle on T2 weight imaging and fluid-attenuated inversion recovery (FLAIR) imaging (**Figures 2C–E**). The lesions were not found with significant enhancement using contrast-enhanced magnetic resonance imaging (**Figure 2F**). Lumbar puncture was also performed, which indicated a slightly increased intracranial pressure (190 mmH_2_O) and normal white cell, total protein, glucose, and chloride levels of the cerebrospinal fluid. Combining the results of the brain MRI and CSF tests, we suggested that the patient has demyelinating diseases of the central nervous system (CNS). We used CBA to detect CNS demyelinating antibodies (AQP4, MOG, and MBP); the CSF and the serum were found to be negative in all antibodies. Moreover, we also search for IgG-oligoclonal bands (OCB) in the CSF and the serum using isoelectric focusing electrophoresis analysis. IgG-OCB was only found in the CSF, and the counts of IgG-OCB were more than two (**Figure 2N**). Furthermore, anti-NMDAR antibodies were detected using the CBA method. According to MacDonald Criteria 2017, the patient was diagnosed as with MS as supported by the clinical feature, dissemination in space demonstrated by MRI, and CSF-specific oligoclonal bands (5). Therefore, the patient was diagnosed as with anti-NMDAR encephalitis overlapping with MS. The patient was on IVMP for 5 days (1,000 mg/day) (the dosage was decreased every 5 days), followed by oral prednisone (60 mg/day) and mycophenolate mofetil (1 g/day). The symptoms of the patient were alleviated after the immunosuppressive treatment. He was continued on immunosuppressive treatment with prednisone (60 mg/day) (the dosage was decreased to be stopped within 1 month) and mycophenolate mofetil (1 g/day) after discharge. On June 26, 2022, the brain MRI result showed that the abnormally high signal lesions were alleviated compared with the prior T2 weight imaging and FLAIR imaging results (**Figures 2G, H**).

On October 1, 2022, the patient was admitted to our hospital again due to a car accident. He was found to have left clavicle and right femoral fractures. The patient decreased the dosage of mycophenolate mofetil (0.5 g/day) by himself since August 2022. A brain MRI was performed again due to the previous clinical history. We found a new lesion with hyperintensity in the left posterior horn of the lateral ventricle on T2 weight imaging and FLAIR imaging (**Figures 2I, J**). The results of all neurological examinations were observed to be in the normal range.

## 3 Diagnostic assessment

The patient’s initial clinical features, such as fever and abnormal behavior, were similar to the presentation of viral encephalomyelitis. The diagnosis of viral encephalomyelitis was supported by abnormal neurological examination results, abnormal imaging findings, and abnormal levels of infection indicators in the blood and CSF. The infection indicators shifted to the normal range after the antiviral treatment, but the symptoms of abnormal behavior were not alleviated. Therefore, we suggested that the patient had developed autoimmune encephalitis after viral encephalomyelitis. Subsequently, we found that the antibodies for autoimmune encephalitis and anti-NMDAR antibodies were positive. This finding confirmed the3diagnosis of anti-NMDAR encephalitis. Then, the immunosuppressive treatment got a good response.

The patient was hospitalized for the second time due to symptoms of dizziness and diplopia. We initially hypothesized that the above-mentioned clinical manifestations were the new symptoms of anti-NMDAR encephalitis. Nevertheless, the findings of the brain MRI, electronystagmography, and CSF tests supported the diagnosis of inflammatory demyelinating disease. Therefore, demyelinating antibodies and IgG-OCB in serum and CSF were used for diagnosis, and the result of IgG-OCB supported the diagnosis of MS. Moreover, anti-NMDAR antibodies were positive in the CSF, but the levels of antibodies were decreased. Hence, the patient was diagnosed as with anti-NMDAR encephalitis overlapping with MS. The mycophenolate mofetil and glucocorticoid immunosuppressive therapy also had positive results. The clinical symptoms vanished throughout the 2 weeks of observation, and the brain MRI results revealed that the lesions had shrunk from their initial size. Nevertheless, the patient’s MRI findings showed a fresh demyelinating lesion at the second follow-up appointment 4 months later.

## 4 Literature review

A literature search was performed using the PubMed and Web of Science database. The following combinations of search terms were used: “multiple sclerosis and anti-N-Methyl-D-Aspartate Receptor encephalitis”, “multiple sclerosis and anti-NMDAR encephalitis”, and “MS and anti-NMDAR encephalitis”. The search was limited to articles in English. A review was done on the information that was available in full-text or abstract form, along with any relevant citations and references. We summarized the clinical manifestations of all 15 cases of overlapping anti-NMADR encephalitis and MS and present them in **Table 1**.

**Table 1:** Characteristics of patients with overlapping anti-NMDAR encephalitis and MS.

Country	Publication date	Sex	Age (years)	Signs of infection	Tumor	Onset	MRI (onset of MS)	MRI (onset of NMDAR)	Immunology findings	Course	Treatment	Prognosis
England (6)	2012	Female	32	Yes	No	Seizures, confusion	Demyelinati-ng changes	Normal	Anti-NMDAR Ab(+), OB(+)	NMDAR to MS	No immunotherapy	Good
Japan (7)	2012	Female	33	Yes	No	Optic nerve involvement	Typical changes with MS	Typical changes with MS	Anti-NMDAR Ab(+), AQP4 (–)	MS to NMDAR	IVMP	Good
Germany (8)	2014	Male	33	No	No	Sensory abnormalities	Typical changes with MS	Typical changes with MS	Anti-NMDAR Ab(+), OB(+)	MS to NMDAR	Plasmapheresis, corticosteroids, mitoxantrone, IVMP	Bad
Austria (9)	2015	Female	26	No	No	Sensory abnormalities	Typical changes with MS	Typical changes with MS	Anti-NMDAR Ab(+), AQP4 (–), MOG (–)	MS to NMDAR	IVMP	NA
Germany (10)	2015	Male	33	No	No	Diplopia	Typical changes with MS	Typical changes with MS	Anti-NMDAR Ab(+), OB(+), AQP4(-)	MS to NMDAR	IVMP, rituximab	NA
England (11)	2017	Female	32	No	No	Seizures, abnormal movement	Typical changes with MS	Demyelinati-ng changes	Anti-NMDAR Ab(+), OB(+), AQP4 (–), MOG (–)	NMDAR to MS	No immunotherapy	Persistent cognitive dysfunction
England (11)	2017	Male	29	No	No	Psychosis, seizures	Demyelinating changes	Demyelinating changes	Anti-NMDAR Ab(+), OB(+)	NMDAR to MS	No immunotherapy	Good
England (12)	2018	Female	41	Yes	No	Visual hallucination, abnormal behavior	Unremarkable	Unremarkable	Anti-NMDAR Ab(+), OB(+)	MS to NMDAR	Plasmapheresis, Rituximab, IVIG	Memory impairment
China (13)	2020	Female	19	No	No	Psychosis, abnormal movement, seizures	Typical changes with MS	Typical changes with MS	Anti-NMDAR Ab(+), OB(+), AQP4 (–), MOG (–)	MS to NMDAR	IVMP. IVIG	Good
Istanbul (14)	2020	Female	26	Yes	No	Sensory abnormalities	Typical changes with MS	Typical changes with MS	Anti-NMDAR Ab(+), OB(+), AQP4 (–), MOG (–)	MS to NMDAR	Plasmapheresis, Rituximab, IVMP, IVIG	Good
Italy (15)	2020	Female	33	No	No	Confusion, psychosis	Typical changes with MS	Typical changes with MS	Anti-NMDAR Ab(+), OB(+), AQP4 (–), MOG (–)	MS to NMDAR	IVMP	Good
China (16)	2021	Female	19	Yes	No	Abnormal movement, sensory abnormalities	Typical changes with MS	Demyelinating changes	Anti-NMDAR Ab(+), OB(+)	NMDAR to MS	IVIG, IVMP, oral glucocorticoid, teriflunomide	Bad
Canada (17)	2021	Female	33	No	No	Confusion, psychosis	Typical changes with MS	Typical changes with MS	Anti-NMDAR Ab(+), anti-ANA(+), anti-ENA(+), anti-RNP(+)	MS to NMDAR	IVMP, IVIG, plasmapheresis	Atypical antipsychotic
England (18)	2021	Male	45	Yes	No	Seizures	Demyelinati-ng changes	Demyelinati-ng changes	Anti-NMDAR Ab(+)	MS to NMDAR	IVIG, IVMP, bortezomib, daclizumab	Good
Iran (19)	2021	Male	34	No	No	Sensory abnormalities	Demyelinati-ng changes	Demyelinati-ng changes	Anti-NMDAR Ab(+), OB(+), AQP4 (–), MOG (–)	MS to NMDAR	Steroids, rituximab	Good
China (our case)	2022	Male	31	Yes	No	Abnormal behavior	Demyelinati-ng changes	Normal	Anti-NMDAR Ab(+), OB(+), AQP4 (–), MOG (–)	NMDAR to MS	IVMP, oral glucocorticoid, mycophenolate mofetil	Good

OB, oligoclonal immunoglobulin bands; MS, multiple sclerosis; NMDAR, N-methyl-D-aspartate receptor encephalitis; anti-NMDAR Ab, anti-N-methyl-D-aspartate receptor antibodies; AQP4, anti-aquaporin 4 antibody; MOG, myelin oligodendrocyte glycoprotein; IVMP, intravenous methylprednisolone; IVIG, intravenous immunoglobulin; NA, not available.

## 5 Discussion

We reported a rare case diagnosed as anti-NMDAR encephalitis after a viral infection, which then progressed to an overlap with MS. Autoantibodies against the receptor’s GluN1 subunit are the main cause of the neuroinflammatory disease known as anti-NMDAR encephalitis. Tumors and herpes simplex encephalitis are two confirmed immunological triggers for anti-NMDAR encephalitis (20). Approximately 64% of patients were detected with anti-NMDAR antibodies within 3 weeks after the onset of herpes simplex encephalitis (20). However, the mechanisms connecting anti-NMDAR encephalitis with MS remain unclear. Some patients have courses from anti-NMDAR encephalitis to MS, but other patients have contrasting courses. Some hypotheses were performed for patients who have courses from anti-NMDAR encephalitis to MS. A previous research reported that NMDAR are present on the myelin sheath formed by oligodendrocytes, and a NMDAR antagonist has a good clinical intervention effect on MS, which suggested that myelin injury resulting from a pathogenic anti-NMDAR antibody burst may cause or exacerbate inflammation in MS (21). In many patients, the NMDAR immune response caused severe infection, and immune disturbance leads to the disruption of immune homeostasis (22), which could be the initiating factor for tissue damage and an inflammatory reaction in MS. There are some other hypotheses for patients who have courses from MS to anti-NMDAR encephalitis according to the current study. As we all know, tumor and viral herpes encephalitis are two confirmed triggers of anti-NMDAR encephalitis (20). Nevertheless, inflammatory demyelination may be a trigger for approximately 3% of patients with anti-NMDAR encephalitis overlapping with demyelinating disorders (23). MS is a typical demyelinating disorder of the central nervous system. Previous studies suggest that MS might expose antigens of the neurons and activate an intrathecal immune response (22, 24).

The diagnosis of this patient is a challenging task for us. In the initial admission, the clinical manifestations, including fever, abnormal behavior, and abnormal infection indicators in blood and CSF, supported the diagnosis of herpes simplex encephalitis. Unfortunately, next-generation sequencing was not performed to detect the herpes simplex virus to verify our hypothesis. The gold standard diagnostic test for anti-NMDAR encephalitis is detected to anti-NMDAR antibodies in serum or CSF of patients (25). Thus, in this study, the patient developed autoimmune encephalitis as evidenced by the anti-NMDAR antibodies found in the serum and CSF. However, the diagnosis of overlapping anti-NMDAR encephalitis and MS has always been a challenge. The clinical manifestations of both overlapping anti-NMDAR encephalitis and MS are highly variable and partly overlapping. MRI, as a common tool to diagnose anti-NMDAR encephalitis and MS, may not demonstrate specific abnormalities to distinguish the two diseases—for instance, demyelinated lesions resembling MS were seen in a case of anti-NMDAR encephalitis (26). In the second admission, the patient showed some new symptoms, such as diplopia and nystagmus. We initially assumed that the symptoms were brought on by a recurrence of anti-NMDAR encephalitis. Nevertheless, the antibody titer (1:10) was lower than the previous (1:30) in CSF. Thus, we assumed that the patient might have a coexisting demyelinating disease. Subsequently, the antibodies of demyelinating diseases were determined to help with the diagnosis, and the CSF was found to be positive with IgG-OCB. Therefore, the patient was diagnosed with overlapping anti-NMDAR encephalitis and MS. In this case, the symptoms in the second admission were not consistent with typical encephalitis, and the possibility of demyelinating diseases could not be ignored. The diagnosis of overlapping anti-NMDAR encephalitis and MS should be based on clinical presentations and laboratory tests, especially in the atypical case.

Overlapping anti-NMDAR encephalitis and MS have been reported in 15 cases. We summarized the clinical features of these patients and present them in **Table 1**, including five from England (6, 11, 12, 18), two from Germany (8, 10), one from Japan (7), one from Austria (9), one from Istanbul (14), one from Italy (15), one from Canada (17), one from Iran (19), and two from China (13, 16). Among the 15 patients, the male-to-female ratio was 2:1. The mean age was 31.2 years. To the best of our knowledge, tumor and viral infections were two confirmed triggers for anti-NMDAR encephalitis. However, no patient was found with tumors, and only six patients showed signs of infections. The symptoms at onset were highly variable. In almost all patients, anti-NMDAR antibodies could be found, and the CSF or serum was positive in IgG-OCB. The MRI result also showed demyelinating lesions. In four patients, the symptoms of anti-NMDAR encephalitis preceded those of MS, while in 11 patients the trajectory was opposite. In general, the coexistence of anti-NMDAR encephalitis and MS was rare, and the clinical features were highly variable.

Up to now, the immunosuppressive therapy of overlapping anti-NMDAR encephalitis and MS has not been systematically studied. In previously reported cases, the most frequently used immunosuppressive therapy was IVMP. Moreover, IVIG, plasmapheresis, and immunosuppressant (*e*.*g*., rituximab, teriflunomide, and bortezomib) were also used in immunosuppressive therapies. However, the curative effects of the above-mentioned immunosuppressive therapies are unclear because two patients had a good prognosis with no immunosuppressive therapy (11). Mycophenolate mofetil is widely used in immunosuppressive therapies for MS (27) or anti-NMDAR encephalitis (28). Therefore, in our case, we first used mycophenolate mofetil for immunosuppressive therapies after the patient was diagnosed with overlapping anti-NMDAR encephalitis and MS. We followed the patient since he received mycophenolate mofetil therapy. The symptoms of anti-NMDAR encephalitis and MS have not reoccurred as of the time that the manuscript was submitted. However, upon performing the brain MRI, a new lesion was found in the third admission, which may have resulted because the patient decreased the dosage of mycophenolate mofetil. The appropriate immunosuppressive therapy for overlapping anti-NMDAR encephalitis and MS needs multicenter randomized controlled trials to be discovered in the future.

In conclusion, we studied a Chinese male patient diagnosed with anti-NMDAR encephalitis who developed MS. MS should be considered when patients with anti-NMDAR encephalitis show atypical symptoms such as diplopia and vertigo, which can be identified through laboratory tests, for CNS demyelinating antibodies and IgG-OCB. Moreover, we were the first to use mycophenolate mofetil for immunosuppressive therapies, which offers a new treatment option for overlapping anti-NMDAR encephalitis and MS.

## Data availability statement

The original contributions presented in the study are included in the article/supplementary material. Further inquiries can be directed to the corresponding author.

## Ethics statement

Written informed consent was obtained from the individual for the publication of any potentially identifiable images or data included in this article. The study protocol was approved by the Ethics Committee and the Expert Committee of The Central Hospital of Shaoyang. The patients/participants provided their written informed consent to participate in this study.

## Author contributions

PL, HZ and YL drafted and revised the manuscript. HL and YL collected the data. CZ designed the study and revised the manuscript. All authors contributed to the article and approved the submitted version.
